# Pearl‐Structure‐Enhanced NASICON Cathode toward Ultrastable Sodium‐Ion Batteries

**DOI:** 10.1002/advs.202301308

**Published:** 2023-04-21

**Authors:** Xin‐Xin Zhao, Wangqin Fu, Hong‐Xia Zhang, Jin‐Zhi Guo, Zhen‐Yi Gu, Xiao‐Tong Wang, Jia‐Lin Yang, Hong‐Yan Lü, Xing‐Long Wu, Edison Huixiang Ang

**Affiliations:** ^1^ Faculty of Chemistry Northeast Normal University 130024 Changchun P. R. China; ^2^ National Institute of Education Singapore Nanyang Technological University Singapore 637616 Singapore Singapore; ^3^ MOE Key Laboratory for UV Light‐Emitting Materials and Technology Northeast Normal University 130024 Changchun P. R. China

**Keywords:** branch chain, high durability, NASICON cathode, sodium‐ion batteries, structural evolution

## Abstract

Based on the favorable ionic conductivity and structural stability, sodium superionic conductor (NASICON) materials especially utilizing multivalent redox reaction of vanadium are one of the most promising cathodes in sodium‐ion batteries (SIBs). To further boost their application in large‐scale energy storage production, a rational strategy is to tailor vanadium with earth‐abundant and cheap elements (such as Fe, Mn), reducing the cost and toxicity of vanadium‐based NASICON materials. Here, the Na_3.05_V_1.03_Fe_0.97_(PO_4_)_3_ (NVFP) is synthesized with highly conductive Ketjen Black (KB) by ball‐milling assisted sol‐gel method. The pearl‐like KB branch chains encircle the NVFP (p‐NVFP), the segregated particles possess promoted overall conductivity, balanced charge, and modulated crystal structure during electrochemical progress. The p‐NVFP obtains significantly enhanced ion diffusion ability and low volume change (2.99%). Meanwhile, it delivers a durable cycling performance (87.7% capacity retention over 5000 cycles at 5 C) in half cells. Surprisingly, the full cells of p‐NVFP reveal a remarkable capability of 84.9 mAh g^−1^ at 20 C with good cycling performance (capacity decay rate is 0.016% per cycle at 2 C). The structure modulation of the p‐NVFP provides a rational design on the superiority of others to be put into practice.

## Graphical abstract

Ketjen Black (KB) branch chains encircle the Na_3.05_V_1.03_Fe_0.97_(PO_4_)_3_ (NVFP) like wearing “pearl,” making the segregated particles link to access the overall conductivity and activating the unusual structural distortion of FeO_6_ in NVFP during electrochemical progress, thus stimulating plenty of Na^+^ de/intercalation at plateau region with reversible phase transition and acquiring a favorable rate performance and durable cycling lifespan under fine volume variation.

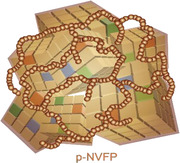

## 1 Introduction

With the robust development of energy storage systems, electric vehicles and electronic devices based on powerful smart grids are also continuously improving, and gradually shifting to the mass market, in turn, bringing more demands for energy storage. Among diversified energy storage systems, devices like rechargeable batteries have become one of the most promising choices for energy reserves with favorable energy conversion efficiency and convenient maintenance.^[^ ^1^ ^]^ At present, rechargeable lithium‐ion batteries (LIBs) have been playing an active role with their strengths such as superior energy density and long service life in commercial applications.^[^ ^2^ ^]^ Nevertheless, developing more promising supplements has been considered an urgent need for boosting grid‐scale electric storage applications in recent decades, as the development of LIBs is severely limited by the limited stock of the raw material and the growing cost. Owing to the widespread abundance and low cost of sodium resources, as well as similar working principles as LIBs, sodium‐ion batteries (SIBs) display great potential in energy storage systems, especially in energy‐intensive environments that focus on economic benefits.^[^ ^3^ ^]^ Nevertheless, the primary challenge of SIBs is to provide efficient electrochemical performance comparable to LIBs, particularly in energy density and cycling stability.

Electrode materials as sodium hosts play a vital role in SIBs, not only as the source of energy but also as the cornerstone of maintaining stability. Commonly, cathode materials of SIBs include layered, polyanionic compounds, Prussian blue and organics^[^ ^4^ ^]^ owing to their intrinsic structural advantages, polyanionic compounds are always regarded as one of the most fascinating cathodes in SIBs: the inductive effect of polyanionic groups makes high operating voltage possible, thereby obtaining relatively high energy density; the robust frameworks are able to diffuse Na^+^ rapidly and provide long‐term structural stability, which is favorable for high power density, operation reliability and security.^[^ ^5^ ^]^ Among the polyanionic compounds, sodium superionic conductor (NASICON) possesses a 3D robust framework, buffering volumetric expansion during phase transition process and strong covalent bonding with predominant ionic conductivity for the migration of Na^+^.^[^ ^6^ ^]^ Eventually, NASICON‐type cathodes like Na_3_V_2_(PO_4_)_3_ (NVP) attract colossal interest, benefitting from their high safety, and high thermal and structural stability.^[^ ^7^ ^]^ NVP has been extensively investigated because of its promising two‐electron reaction, high sodium ion mobility, high working voltage plateau of about 3.4 V, bringing it into the limelight of large‐scale energy storage.^[^ ^4^, ^8^ ^]^ However, the toxicity and cost of vanadium have become the bottleneck which limit its practical application.

Thanks to the flexible tunability of the NASICON structure, different choices of transition metal sites have been verified by the replacement of V with various elements in NVP.^[^ ^9^ ^]^ The regulation of Mn^3+^/Mn^2+^, Fe^3+^/Fe^2+^ and Ti^3+^/Ti^2+^ redox centers has brought a series of cathode materials, which show promising features in terms of enhanced output voltage and specific capacity.^[^ ^9^, ^10^ ^]^ The iron‐based compounds in V‐substituted cathodes have better cycling stability compared with Mn‐based counterparts with the Jahn‐Teller effect and possess a higher redox potential of Fe^2+^/Fe^3+^ (2.4 V) than that of Ti‐based substitution materials (Ti^3+^/Ti^4+^ (2.1 V)). Therefore, in the wide selection of transition metals, the choice of Fe substitution of the V site is considered to be the best choice for reducing toxicity and cost compared to NVP as well as maintaining fine electrochemical properties.^[^ ^4^, ^6^, ^7^, ^11^ ^]^

Considering more accumulation of Na^+^ and reducing the content of vanadium, Na_4_Fe^II^V^III^(PO_4_)_3_ emerges in the class of mixed‐metal phosphates, which is expected to display great performance through the successive activations of the Fe^3+^/Fe^2+^, V^4+^/V^3+^ and V^5+^/V^4+^ redox couples.^[^ ^6^, ^12^ ^]^ Although the systematic study and attempts have been implemented in preparing Na_4_VFe(PO_4_)_3_ directly, the achievements often fall short of expectations, tending to form the sodium‐deficient composition with mixed valence Fe^2+^/Fe^3+^ (e.g., Na_3.41_£_0.59_FeV(PO_4_)_3_ and Na_3.36_FeV(PO_4_)_3_ via a traditional sol‐gel route).^[^ ^6^, ^9^ ^]^ The absence of Na^+^ in the sodium‐deficient composition needs to be compensated by the free Na^+^ in the electrolyte to obtain the Na_4_VFe(PO_4_)_3_ with electrochemical cycles. What's more, a systematic study of Na_3+_ *_x_* Fe*_x_* ^II^V_2‐_ *_x_* ^III^(PO_4_)_3_ is also to produce secondary phases (mainly NaFePO_4_) when *x* > 0.5 in solid‐state synthesis. It is difficult to prepare a pure‐phase Na_4_VFe(PO_4_)_3_ target as many secondary phases appeared in the samples through direct synthesis. Recently, an effective way was implemented by ball‐milling assisted sol‐gel method combined with heat treatment in preparing the pure‐phase Na_4_VFe(PO_4_)_3_. In such a synthesis process, the amorphous carbon single layer coated on the surface of Na_4_VFe(PO_4_)_3_ was only with 3 nm thickness and assist Na_4_VFe(PO_4_)_3_ to obtain a typical rhombohedral NASICON‐type structure without other impurities. The thin carbon layer from the carbonization of glucose not only improves the electronic conductivity of Na_4_VFe(PO_4_)_3_ materials intrinsically but also influences crystal structure to ensure the stable state of Fe in Na_4_VFe(PO_4_)_3_. In addition, a handy way is using Na_3_Fe^III^V^III^(PO_4_)_3_ as a precursor by electrochemical synthesis to obtain pure Na_4_Fe^II^V^III^(PO_4_)_3_. And the Na_3_FeV(PO_4_)_3_ itself is easy to synthesize and has a decent electrochemical performance with two voltage plateaus at 3.3 and 2.5 V.^[^ ^6c^ ^]^ Despite the local environments and valence states of Fe and V being different in Na_3_FeV(PO_4_)_3_ and Na_4_VFe(PO_4_)_3_, they similarly deliver an initial discharge capacity of about 100 mAh g^−1^ based on a two‐electron reaction, and Na_3_FeV(PO_4_)_3_ shows a durable capacity retention even at 10 C with subtle volume change. Therefore, Na_3_FeV(PO_4_)_3_ has advantages in terms of manufacturing technique, specific capacity and cycle durability compared with Na_4_VFe(PO_4_)_3_ based on the V^4+^/V^3+^ and Fe^3+^/Fe^2+^ redox reaction.

Herein, in this work, we engineer an extraordinary NASICON material Na_3.05_V_1.03_Fe_0.97_(PO_4_)_3_ (NVFP) by partially replacing V with Fe. Moreover, besides the carbon coating produced from the sol‐gel process, the spherical highly conductive Ketjen Black (KB) with a unique shape of branched chains is also selected to improve the electronic conductivity, balance the valence state and modulate the main structure in the initial electrochemical process. The branched chains like “pearl” encircling independent NVFP particles (p‐NVFP) provide more conductive paths, greatly improving the current density and battery capacity, and further extending the service time of the battery. The particular structure of p‐NVFP presents the performance with favorable capacity (106.8 mAh g^−1^ at 0.2 C), remarkable high‐rate capacity (87.8 mAh g^−1^ even at 15 C) and long cycling (stable operation exceeds 5000 cycles at 5 C). Furthermore, we elucidate the effects of active branch chain infusing on the crystal structure distortion by using in situ X‐ray diffraction (XRD) and ex situ X‐ray photoelectron spectroscopy (XPS). The ionic migration kinetics in such a special structure is to be investigated by the steady operation of p‐NVFP by cyclic voltammetry (CV) curves and galvanostatic intermittent titration technique (GITT). Stimulating from the rapid kinetics at voltage plateaux, the full cells of p‐NVFP reveal a remarkable capability with high capacity retention of 84.9 mAh g^−1^ at 20 C and durable cycling performance (capacity decay rate is with 0.016% per cycle at 2 C). Such a simple but effective structure design buffers the impact of current on the host materials by the decentralized transmission network of “pearl,” and mixed state of p‐NVFP makes the solid‐solution reaction adequate in sodium storage mechanism, which contributes a great insight for other systems with cost‐effective elements for large‐scale energy storage applications.

## 2 Result and Discussion

### 2.1 The Morphological and Structural Characterizations of p‐NVFP

**Figure 1:**
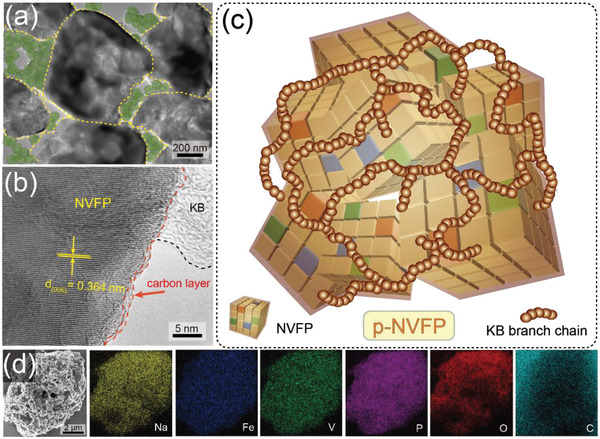
a,b) TEM and HRTEM images of p‐NVFP. c) Schematic diagram of p‐NVFP. d) EDX element distribution of the p‐NVFP material.

**Figure 2:**
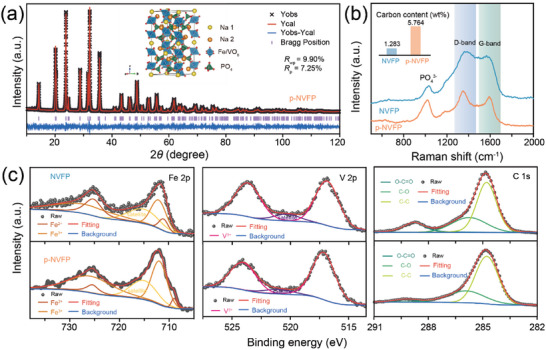
a) Observed and calculated XRD patterns of p‐NVFP. b) Raman spectra of the NVFP‐based materials (the inset is carbon content). c) High‐resolution XPS spectra and the corresponding deconvolution results for the orbital peaks of Fe 2p, V 2p, C 1s of NVFP‐based materials.

The p‐NVFP particles were synthesized via ball‐milling assisted sol‐gel method in the Experimental Section. The same production process was used in preparing NVFP (without joint of KB) and p‐NVFP (design with branch chains like “pearl” of KB). The morphology and microstructure of NVFP‐based materials were monitored by scanning electron microscopy (SEM) and transmission electron microscopy (TEM) in Figures S1 and S2, Supporting Information. Irregular primary particles with a size of 50–500 nm tend to stack up into secondary particles at the micron‐sized level with coating layers, which is related to the citric acid pyrolysis in the sol‐gel process thus limiting the growth of particles. Besides, the branch chains of spherical KB adhere to the edge of the particles grain like “pearl” in TEM images (**Figure** **1**a and Figure S1b, Supporting Information), making the segregated NVFP particles link to each other and accessing the overall conductivity. Furthermore, the HRTEM image (Figure 1b) of p‐NVFP not only displays obvious lattice fringes whose width of 0.364 nm corresponds to the (006) planes of NVFP but also exhibits the amorphous carbon layers and the joint of KB particles. Because of this, more NVFP particles with carbon pyrolysis coating are activated during the random extension of KB branch chains as Figure 1c shown. Furthermore, the energy dispersive X‐ray spectrometer (EDX) mapping diagrams (**Figure** **2**e and Figure S2, Supporting Information) exhibit the uniform distribution of Na, Fe, V, P, O elements in p‐NVFP and NVFP particles and a clear difference in the C element from the additional KB. Therefore, the structure with multiple electronic pathways has been realized with special branch chains like “pearl” in p‐NVFP.

As shown in Figure S3, Supporting Information, the XRD patterns of NVFP and p‐NVFP are consistent with the same structure and belong to the same space group *R*_3c with NVP.^[^ ^6^, ^13^ ^]^ While there is a trace of undesirable NaFePO_4_ in NVFP like in the previous research, it disappears after ball‐milling with KB followed by annealing at high temperature. And the main phase NVFP is consistent with the standard card of NVP (PDF#00‐062‐0345). Simultaneously, the crystal structure of p‐NVFP is derived from the Rietveld refinement of the XRD as illustrated in Figure 2a. The calculated pattern is in remarkably great agreement with the collected profile, demonstrating a great indexed result of synthesized p‐NVFP with typical trigonal NASICON‐type structures.^[^ ^7^, ^11^, ^14^ ^]^ The octahedra are randomly filled by Fe and V ions. The Fe/VO_6_ octahedra connected with PO_4_ tetrahedra by corner‐sharing, establishing a 3D anion structure for accommodating Na^+^ flux. With different oxygen environments, Na^+^ at two differentiated sites possess different coordination in the interstitial space. Analyzed by Raman spectroscopy, two characteristic carbon peaks for NVFP‐based materials, which were located at about 1380 cm^−1^ and 1590 cm^−1^, corresponding to the disordered carbon and graphitized carbon (D band and G band), respectively.^[^ ^3^, ^10^, ^14^ ^]^ And the graphitized proportion is measured by the relative intensity of two (*I*_D_/*I*_G_), which originated from carbon components (citric acid and KB). The *I*_D_/*I*_G_ values of NVFP, p‐NVFP are 1.33 and 1.01, respectively. Moreover, the carbon contents of NVFP and p‐NVFP materials are 1.28 wt% and 5.76 wt%, measured with the elemental analysis are shown in Figure 2b inset, which is consistent with KB introduction with a slight loss. The residual KB particles in p‐NVFP offer favorable electronic conductivity, which is 4.85×10^−11^ S cm^−1^ compared to 6.37 × 10^−12^ S cm^−1^ of NVFP according to Table S1, Supporting Information. The results reveal that conductivity is increased eight times by introducing 4 wt% of KB. And the surface elemental compositions of NVFP‐based materials were further investigated by XPS in Figure 2c. Three same types of carbon signals in the C 1s spectrum of the two, which belong to the C—C, C—O, and O—C=O bonds in the carbon components.^[^ ^15^ ^]^ The spectrum of V 2p splits into two sharp peaks located at about 523.42 eV and 516.40 eV, corresponding to the electrons in V 2p_1/2_ and V 2p_3/2_, respectively, confirming trivalent V and in good agreement as expected.^[^ ^16^ ^]^ The high‐resolution Fe 2p XPS spectra of p‐NVFP are split into five peaks with one satellite peak, two pairs of sharp peaks centered at about 711.96 eV and 726.45 eV, 709.6 eV and 725.69 eV, corresponding to the electrons in Fe^3+^ and Fe^2+^, respectively, confirming trivalent and partly divalent in the p‐NVFP.^[^ ^17^ ^]^ The molar ratio of compounds in p‐NVFP is 3.05: 0.97: 1.03 for Na, Fe and V by inductively coupled plasma spectroscopy (ICP‐OES) chemical analysis, which is well matched for the mixed state of Fe. Finally, based on all the above results, it can be inferred that the p‐NVFP material is a mixed state Fe‐substituted phosphate compound for NVP.

### 2.2 Electrochemical Performance of p‐NVFP

**Figure 3:**
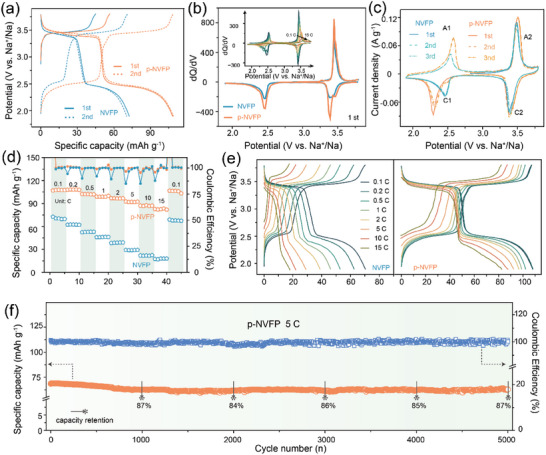
a) The first two GCD curves at 0.1 C. b) d*Q*/d*V* curves of NVFP and p‐NVFP half cells in the first 5 cycles, the inset is corresponding curves at different rates. c) The initial three CV curves at a scan rate of 0.1 mV s^−1^. d) The rate performance of NVFP and p‐NVFP half cells. e) The GCD curves at different rates from 0.1 to 15 C. f) Cycling stability at 5 C of p‐NVFP half cells.

To explore the Na storage ability of NVFP‐based materials, the electrochemical tests were implemented with Na plate as a reference electrode in the voltage window of 1.9–3.8 V. And the first two galvanostatic charge/discharge (GCD) curves of NVFP‐based materials were compared in **Figure** **3**a. The initial charge process has only V^3+^/V^4+^ redox reaction at high voltage plateau and takes off more Na^+^ in p‐NVFP than those in NVFP, thanks to the collection toward current of KB branch chain. As a result, the capacity of p‐NVFP cells reaches 106.8 mAh g^−1^ in first discharge progress and the initial Coulombic efficiency (CE) of the two is above 100%, which corresponds to consuming some Na^+^ in electrolyte. However, the CE of the two remains around 100%, without continuously consuming additional Na^+^ during subsequent cycles at the same current. And the d*Q*/d*V* comparison also demonstrates that the reduction peaks of p‐NVFP are higher than the counterpart around 3.45 V of NVFP. Similarly, the anodic peak (A2) at high plateau in the first cycle is sharper than other anodic peaks and located at higher potential (3.50 V), originating from the adequate V oxidation during Na^+^ de/intercalation in CV curves of Figure 3c. Meanwhile, the cathodic peak (C2) subsequently of p‐NVFP also showed a clear shift of peak positions (3.38 V) in the first discharge process, which is pretty much the same as that in NVFP (3.37 V). In addition, the cathodic peak (C1) of p‐NVFP in the subsequent cycles presents two peaks (2.49 and 2.32 V), differing from those of NVFP (around 2.46 V). The voltage differences are inextricably linked to the state of redox reactions of V/Fe, rooting from Na^+^ migration around the Fe/VO_6_ octahedra under the influence of “pearl” structure.^[^ ^18^ ^]^ Except for the first cycle, the respective CV curves coincide well in the subsequent cycles, implying the stable effect of the two on the reversible structure. Such a differentiation would make an effect on the subsequent electrochemical processes, which will be discussed in the following section.

In addition, the potential differentiation of p‐NVFP also exists in the initial cycling according to Figure 3d,e. The rate performance of the p‐NVFP composites lefts that of NVFP far behind as expected in Figure 3d, benefitting from improved electronic conductivity and structure optimization. Even at a high rate of 15 C, the specific discharge capacity of p‐NVFP was still above 76% of the initial capacity, which is far better than that of NVFP (only 18 mAh g^−1^). The revert capacity up to 105.7 mAh g^−1^ was obtained when the current density returned to 0.1 C. After several cycles at 0.1 C, the rate performance at 0.2 C elevated slightly and reached 108.2 mAh g^−1^. Admittedly, the voltage differentiation induced by “pearl” structure makes the polarization obviously decrease, especially at high current density in Figure 3e. The redox couple not only de/intercalates Na^+^ adequately even in fast charge process, but also maintains stable intercalation reaction, thus the electrochemical property of p‐NVFP is superior to NVFP even with the same redox reaction. Benefitting from the improved reversibility, the cycling stability of p‐NVFP also gets promoted as shown in Figure 3f. The p‐NVFP half cells exhibit an outstanding capacity retention rate of 87.7% at 5 C after 5000 cycles. Therefore, the p‐NVFP produces the voltage differentiation for electrochemical reaction, which is worthy of further research on “pearl” structure using other means.

### 2.3 Sodium Storage Mechanism and Structural Evolution of p‐NVFP

**Figure 4:**
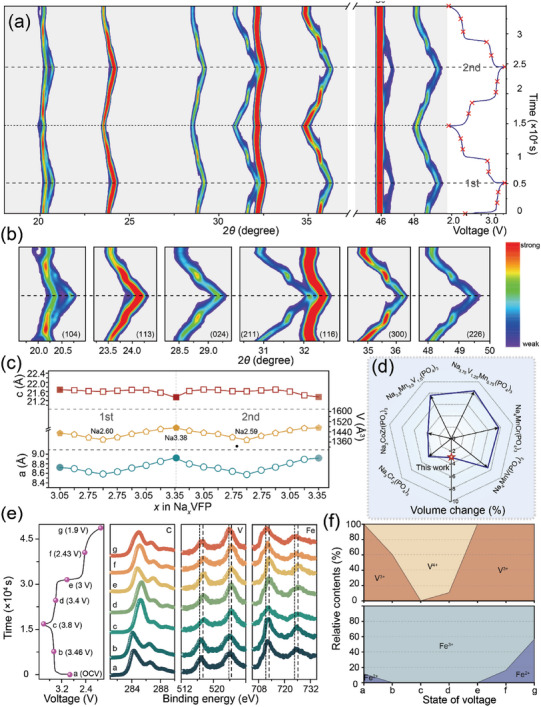
a) In situ XRD patterns of p‐NVFP half cells. b) The corresponding enlarged images of the specific angles of the second cycles. c) The lattice parameters variation during the first two cycles. d) The volume change comparison of common NASICON materials. e) The ex situ XPS variation of the first cycle. f) The relative contents of V and Fe at different states of voltage.

Utilizing the in situ XRD and ex situ XPS technologies, the sodium storage mechanism of p‐NVFP in the initial two cycles was elaborated in **Figure** **4**. The evolution is observed during the two cycles: all the diffraction peaks can be indexed to the NASICON‐structural NVFP phase in Figure 4a. According to the patterns in Figure 4b, the diffraction peaks shift toward higher angles during the charge process and come back to lower angles monotonically in the discharge process.^[^ ^19^ ^]^ All the XRD peaks almost return to their original Bragg positions and intensities after one charging/discharging cycle, indicating excellent reversibility of insertion/extraction with a single‐phase solid solution reaction.^[^ ^20^ ^]^ Figure 4c illustrates the lattice parameters calculated from selected patterns (specific variable amounts of Na^+^) for the first two cycles. The previous research showed that there was a continuous decrease in the lattice parameter a, while the c parameter increased during the charge process.^[^ ^1^, ^10^ ^]^ However, the lattice parameter *c* goes down in the first charge process and rises first and then decreases in the second charge process, which seems to produce unusual structural distortion compared to NVP. The structure evolution goes further with the shrink of subsequent transport channels along c axis, thus making more Na^+^ extract from the main structure, which corresponds to more capacity at high voltage plateau in charge process. It is speculated that the peculiar decrease of *c* parameter could be likely to relate to the contraction of FeO_6_ octahedrons with the oxidation of Fe^2+^ to Fe^3+^. The changes in valence‐state and relative contents of Fe and V provide direct evidence to comprehend the redox process of the p‐NVFP during charging/discharging process. As shown in Figure 4e and Figures S4 and S5, Supporting Information, the ex situ XPS spectra are collected for various state of voltage of p‐NVFP.

Before the potential of V^3+^/V^4+^ at 3.45 V, the trace of Fe^2+^ in p‐NVFP would generate surface oxidation to Fe^3+^ (peaks at 725.48 and 711.58 eV reveal the characteristic of Fe^2+^ and peaks at 726.18 and 712.18 eV reveal the characteristic of Fe^3+^). At the same time, the XPS spectra of C1s at point b (3.46 V) demonstrate a shift toward low binding energy, differing from oxidation in charge process and implying the interaction with FeO_6_. The valence‐state changes between point a (open circuit voltage, OCV) and point b (3.46 V) in Figure 4e, Fe are corresponding to the decrease of c value in Figure 4c, which reveals the superiority of “pearl” structure on the oxidation of Fe^2+^ in p‐NVFP along with the structural adjustment. Through this modulation, p‐NVFP obtains the preferable buffer ability of Na^+^, whose volume change is significantly lower than other NASICON materials in Figure 4d.^[^ ^4^, ^9^, ^21^ ^]^ In summary, the “pearl” structure makes the Fe^2+^ of NVFP take surface oxidation, assisting more deintercalation of Na^+^ along c axis in the initial structural adjustment and stimulating the subsequent V^3+^/V^4+^ redox completely.

### 2.4 Diffusion Kinetics of Na^+^ and Full Cell Behavior

**Figure 5:**
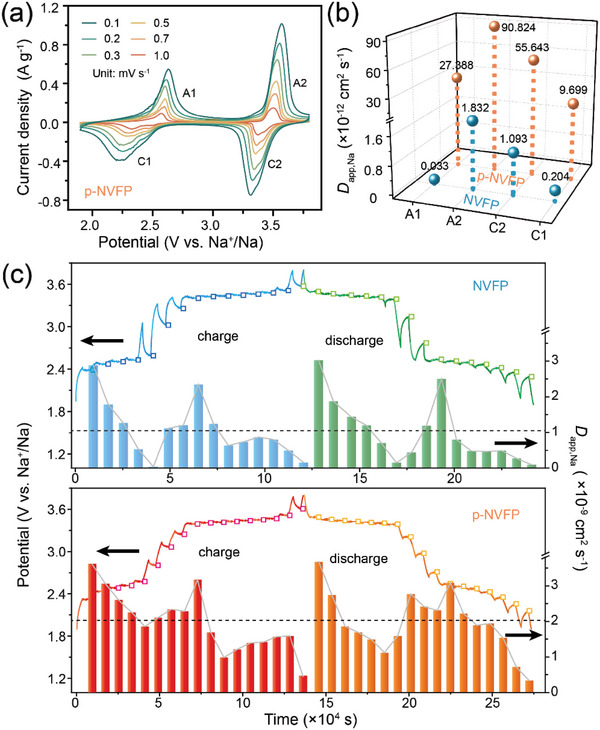
a) CV curves at various scan rates for p‐NVFP. The *D*_app,Na_ comparison calculated b) by CV tests; c) along with time by GITT tests. The line is the GCD curves during GITT tests and the symbol is the quasi‐equilibrium potential for each relaxation process.

Furthermore, the kinetic process of p‐NVFP with voltage differentiation was performed to study the electrode reaction by CV tests at different sweep rates and GITT. As shown in **Figure** **5**a and Figure S6, Supporting Information, the CV curves of NVFP‐based electrode at sweep rates of 0.1–1.0 mV s^−1^. Figure S6 (Supporting Information) displays the linear fitting of the peak currents (*I*_p_) as a function of scan rates square root (𝜐^1/2^), suggesting that the Na^+^ extraction/insertion mechanism is controlled by the diffusion process. Calculated by CV profiles, the *D*_app,Na_ values of p‐NVFP are about tens to hundreds of times higher than those of NVFP at respective redox reactions in Figure 5b, which manifest the rapid diffusion kinetics during Na^+^ de/intercalation with improved performance by “pearl” structure. Figure 5c shows the curve of the galvanostatic time (*τ*) and the corresponding cell voltage (*E*) for electrode material during the second cycle. And the fitting results between *E* and the square root of galvanostatic time (*τ* ^1/2^) during a single GITT titration process are shown in Figure S6, Supporting Information. Generally, the kinetics of the de/intercalation process in the plateau region was the rate‐determining step of electrodes. And all the *D*_app,Na_ values of p‐NVFP in the voltage plateau region are also higher than those of the NVFP electrode, which is consistent with the results of the CV tests. In particular, the diffusion at the low plateau region of Fe^2+^/Fe^3+^ in NVFP is far below the average *D*_app,Na_ value (dotted lines in each picture) and significantly behind p‐NVFP (the average *D*_app,Na_ value of NVFP and p‐NVFP is 1.07 × 10^−9^ and 2.01 × 10^−9^ cm^2^ s^−1^, respectively). In addition, the *D*_app,Na_ value drops down markedly between the plateau regions in NVFP, which is obviously different from p‐NVFP, whether is in charging/discharging process. The reduction of the difference is related to the unusual structural distortion in p‐NVFP as mentioned in Figure 4, which promotes more significantly in the *D*_app,Na_ value of low potential plateaux. Hence, the excellent kinetic properties further explain the reasons for the improved electrochemical performance and cycle performance, which are caused by the modulation of branch chains in the “pearl” structure.

**Figure 6:**
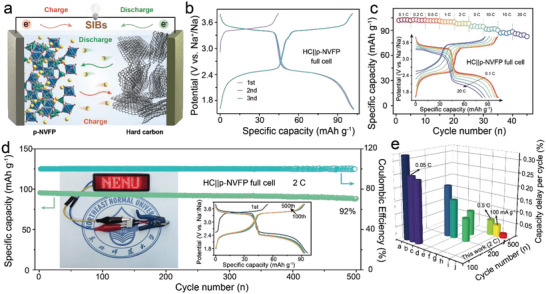
a) Schematic illustration of the sodium‐ion full‐cell with p‐NVFP cathode and hard carbon anode. b) Charging/discharging profile of the first three cycles for HC||p‐NVFP full cells. c) The rate performance and corresponding GCD curves of HC||p‐NVFP. d) The cycling ability of the full cells, the left inset photograph is LED gramps lighted up with the full cell and the right inset is GCD curves of different cycles. e) The capacity delay comparison of the full cells in SIBs (The current density is 1 C if not specified).

Based on the above results, the full cells using hard carbon (HC) as anode were assembled and the capacity was controlled by the cathode (N/P = 1.1), schematically illustrated in **Figure** **6**a. The amorphous HC was purchased commercially without further processing and its structure is characterized in Figure S8a (Supporting Information). HC electrode was prepared in the same manner as p‐NVPF electrode. Note that, the HC electrodes have been activated before assembling and their electrochemical performance in half cell is shown in Figure S8b, Supporting Information, which reveals highly stable cycling performance, hence the initial discharge capacity of the full cells achieves 102.5 mAh g^−1^ at 0.1 C and retains 84.9 mAh g^−1^ even at 20 C in Figure 6b,c.^[^ ^22^ ^]^ Impressively, the cycling stability of the full‐cell is as excellent as that of the p‐NVFP half cell in Figure 6d, which exhibits an initial discharge capacity of 95.7 mAh g^−1^, and delivers capacity retention of 92% after 500 cycles at 2 C and the capacity decay rate reaches to 1.5‱ per cycle. Figure 6e summarizes the electrochemical performance of recently reported sodium‐ion full‐cell (detailed parameters are listed in Table S2, Supporting Information). In comparison, p‐NVFP||HC full cells display great prospects for practical utilization, in terms of large reversible capacity and stable service lifespan.

## 3 Conclusion

The effects of active branch chain infusing on the crystal structure distortion were analyzed by in situ XRD and ex situ XPS, and ionic migration kinetics were examined by CV curves and GITT to have an insight into the structure modulation of p‐NVFP. The branch chains like “pearl” encircling independent p‐NVFP provide more conductive paths, greatly improving the current density and battery capacity, and further extending the service time of the battery. The particular structure of p‐NVFP presents the performance with favorable capacity (106.8 mAh g^−1^ at 0.2 C), remarkable high‐rate capacity (87.8 mAh g^−1^ even at 15 C) and long cycling (stable operation exceeds 5000 cycles at 5 C). Stimulating from the rapid kinetics at voltage plateaux, the full cells were assembled with the commercial HC. p‐NVFP reveals a remarkable capability with a high capacity of 84.9 mAh g^−1^ at 20 C and durable cycling performance (capacity decay rate is 0.016% per cycle at 2 C). Such a simple but effective structure design buffers the impact of current on the host materials and makes the solid‐solution reaction adequate in sodium storage mechanism, which contributes a great insight for other cost‐effective systems for large‐scale and durable cycling lifespan in energy storage applications.

## 4 Experimental Section

### Preparation of NVFP and p‐NVFP Material

The NVFP cathode materials were synthesized by a sol‐gel method. First of all, a stoichiometric amount of Fe(NO_3_)_3_∙9H_2_O, NH_4_VO_3_, and CH_3_COONa were added with a certain amount of distilled water into the round‐bottomed flask. Then, a saturated citric acid solution was added dropwise to the round‐bottomed flask as carbon source and complex agent and stirred for 30–50 min to make the solution evenly mixed. And then added NH_4_H_2_PO_4_ into the above solution while stirring. Subsequently, the distilled water was evaporated at 80 °C to form a wet gel. The resulting wet gel was dried at 100 °C overnight in a vacuum oven to form a dry gel. After grinding the dry gel, it was pre‐calcined at 350 °C for 4 h in the reducing atmosphere. The samples after pre‐calcination were ball‐milled and calcined at 500 °C for 8 h to obtain the final product NVFP. The synthetic raw material is denoted as NVFP, and the ball‐mill product with adding 4wt% KB is denoted as p‐NVFP.

### Materials Characterization

The X‐ray diffraction (XRD) dates were collected in an X‐ray diffractometer (Bruker D8 Advance, Germany) with Cu K_*α*_ radiation (*λ* = 0.15418 nm) in the scan range (2*θ*) of 10–80°. The morphologies information, element distribution and particle size of each compound were obtained by using the scanning electron microscope (SEM, Hitachi SU8000) and high‐resolution transmission electron microscopy (HR‐TEM, JEOL‐2100F, 200 kV) equipped with an energy dispersive X‐ray spectrometer (EDX). The Raman spectra were obtained by using a laser confocal micro‐Raman spectrometer (JYHR‐800 Lab Ram) with an excitation laser beam wavelength of 488 nm. The X‐ray photoelectron spectroscopy (XPS) analysis was obtained by ESCALab 220i‐XL electron spectrometer from VG Scientific with 300 W Al K_*α*_ radiation to determine the valence states of the prepared materials. And the carbon content was measured by CHN Elemental analyzer (Eurovector‐EA3000). Inductively coupled plasma spectroscopy (ICP) analysis was obtained by Prodigy. Electronic conductivity testing was measured by four‐terminal powder resistivity tester (ST2722‐SZ).

### Electrochemical Measurements

The cathode electrode consisted of the active material NVFP, the conductive agent KB and the binder carboxymethylcellulose (CMC) with a mass ratio of 8:1:1. The slurry was uniformly coated on the Al foil. The electrodes were dried in a vacuum oven at 60 °C for 10 h. The loading mass of active material on the final working electrode is about 0.8–1.3 mg cm^−2^. The CR2032 type coin cells consisted of the prepared electrode as the cathode, the metal sodium plate as the counter and reference electrode, the glass microfiber membrane (Whatman) as the separator, and electrolytes (1 mol L^−1^ NaClO_4_ dissolved in the ethylene carbonate (EC) and propylene carbonate (PC) solution with a volume ratio of 1:1 and 5 vt% fluoroethylene carbonate (FEC) as an additive) were assembled. The galvanostatic charge/discharge (GCD) tests at different current densities were implemented on the battery testing systems (NEWARE CT‐4000). Cyclic voltammetry (CV) tests were obtained on a Versa STAT 3 electrochemical workstation in the potential range of 1.9–3.8 V. The current density of the galvanostatic intermittent titration technique (GITT) tests was 0.05 C in the potential range of 1.9–3.8 V, the duration time of each current pulse was 1 h, and the resting time was 6 h. The p‐NVFP cathode was matched with the hard carbon (HC) anode in a glove box to assemble full cells, and their electrochemical performance was tested within the same potential window as half cells. The specific capacity of the full cell was calculated based on the mass of the active material in the cathode. Before assembling the full cells, presodiation for the HC anode was performed to activate the material and stabilize the electrode surface. Specifically, by chemical presodiation, electrolyte was added to the anode material and contacted with the metal sodium directly for 30 min.

### Calculation of Diffusion Kinetics

The fitted relationship between the peak current density (*i*_p_) and the square root of the sweep rate (*v* ^1/2^) derived from the CV curves. Of all the NVFP‐based composites can observe a good linear fitting result clearly, proving that de/intercalation of Na^+^ during the charge‐discharge process is controlled by diffusion. Therefore, the corresponding (*D*_app, Na_) in the electrode material can be calculated by the following Randles‐Sevcik formula:

(1) $$i_{p}\;=\;2.69\times 10^{5}n^{3/2}AD_{\mathrm{app},\mathrm{Na}}^{1/2}C_{0}v^{1/2}$$

where *i*_p_ is the peak current density, *n* is the number of electrons transferred per reactant molecule during the charge‐discharge process of the NVFP electrodes (*n* = 2 for the NVFP phase), *A* is the electrode surface area of 1.13 cm^2^, and *C*_0_ is the concentration of Na ion in the crystal, and *v* is the sweep rate.

The apparent sodium ion diffusion coefficients (*D*_app,Na_) were further calculated by GITT tests to evaluate the sodium ion diffusion kinetics of the three NVFP‐based composites. The *D*_app,Na_ can be calculated by the following formula:^[^ ^3b^ ^]^

(2) $$D_{\mathrm{app},\mathrm{Na}}\;=\;\frac{4}{\pi \tau }{\left(\frac{m_{B}V_{M}}{M_{B}S}\right)}^{2}{\left(\frac{\Delta E_{s}}{\Delta E_{\tau }}\right)}^{2}\;\left(\tau \;\ll \;L^{2}/D\right)$$

where *m*_B_ is the mass of the NVFP material, *V*_M_ is the molar volume, *M*_B_ is the molar molecular mass, *τ* is the time for an applied galvanostatic current (3600 s) during each titration, *S* is the electrode surface area (1.13 cm^2^), and *L* is the average radius of the material particles, Δ*E*_s_ and Δ*E*_*τ*_ are the quasi‐equilibrium potential and the change in cell voltage during the current pulse, respectively.

## Conflict of Interest

The authors declare no conflict of interest.

## Supporting information

Supporting Information

## Data Availability

Research data are not shared.
