# Supplementary material for: Pearl‐Structure‐Enhanced NASICON Cathode toward Ultrastable Sodium‐Ion Batteries
Source: Adv Sci (Weinh). 2023 Apr 21;10(19):2301308. doi: 10.1002/advs.202301308 (PMC10323620; doi:10.1002/advs.202301308)
Supplement: Supplementary file 1 — Supporting Information [file ADVS-10-2301308-s001.pdf]

ADVANCED SCIENCE
Open Access

Supporting Information

for *Adv. Sci.*, DOI 10.1002/advs.202301308

Pearl-Structure-Enhanced NASICON Cathode toward Ultrastable Sodium-Ion Batteries

Xin-Xin Zhao, Wangqin Fu, Hong-Xia Zhang, Jin-Zhi Guo, Zhen-Yi Gu, Xiao-Tong Wang, Jia-Lin Yang, Hong-Yan Lü, Xing-Long Wu* and Edison Huixiang Ang**

Supporting Information

Pearl-Structure-Enhanced NASICON Cathode Towards Ultrastable Sodium-Ion Batteries

Xin-Xin Zhao[1], *Wangqin Fu*[1], *Hong-Xia Zhang, Jin-Zhi Guo*, Zhen-Yi Gu, Xiao-Tong Wang, Jia-Lin Yang, Hong-Yan Lü, Xing-Long Wu*, and Edison Huixiang Ang**

X.-X. Zhao, H.-X Zhang, Dr. H.-Y. Lü, Prof. X.-L. Wu

Faculty of Chemistry

Northeast Normal University

Changchun 130024, P. R. China

Email address: xinglong@nenu.edu.cn

W. Fu, Prof. E. H. Ang

National Institute of Education Singapore,

Nanyang Technological University Singapore,

637616, Singapore

Email: edison.ang@nie.edu.sg

Dr. J.-Z. Guo, Z.-Y. Gu, X.-T. Wang, J.-L. Yang, Prof. X.-L. Wu

MOE Key Laboratory for UV Light-Emitting Materials and Technology

Northeast Normal University

Changchun 130024, P. R. China

Email address: guojz065@nenu.edu.cn

[1] These two authors contributed equally to this work.

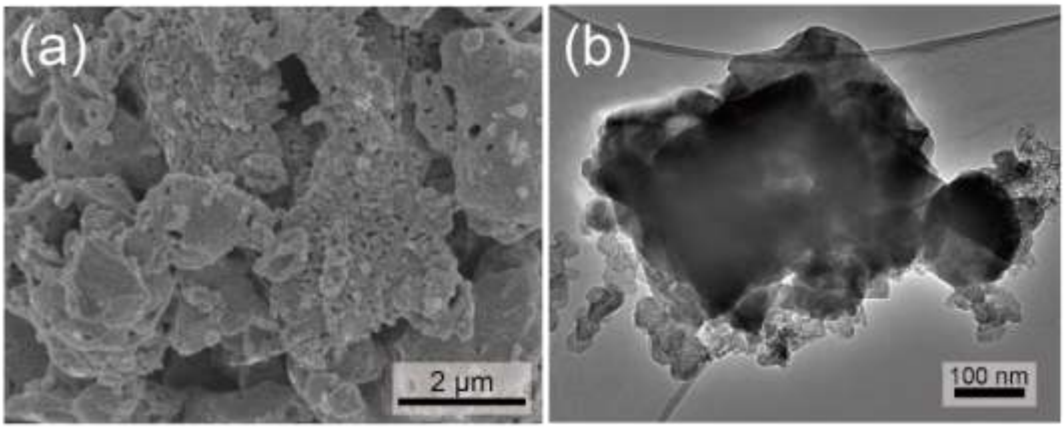

Figure S1. (a) SEM image and (b)TEM image of p-NVFP.

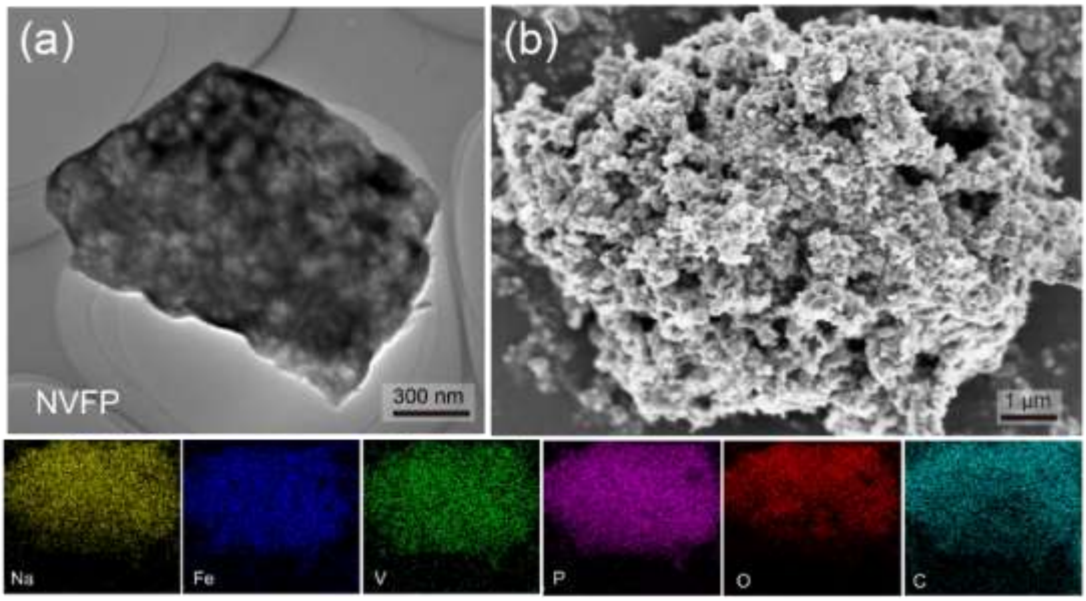

Figure S2. (a) TEM image and (b) SEM image and the corresponding elemental EDX mappings of NVFP.

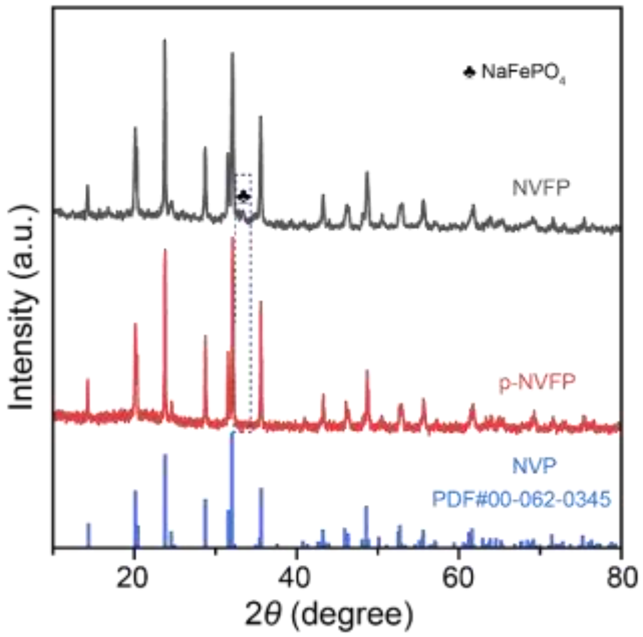

Figure S3. XRD of NVFP-based material.

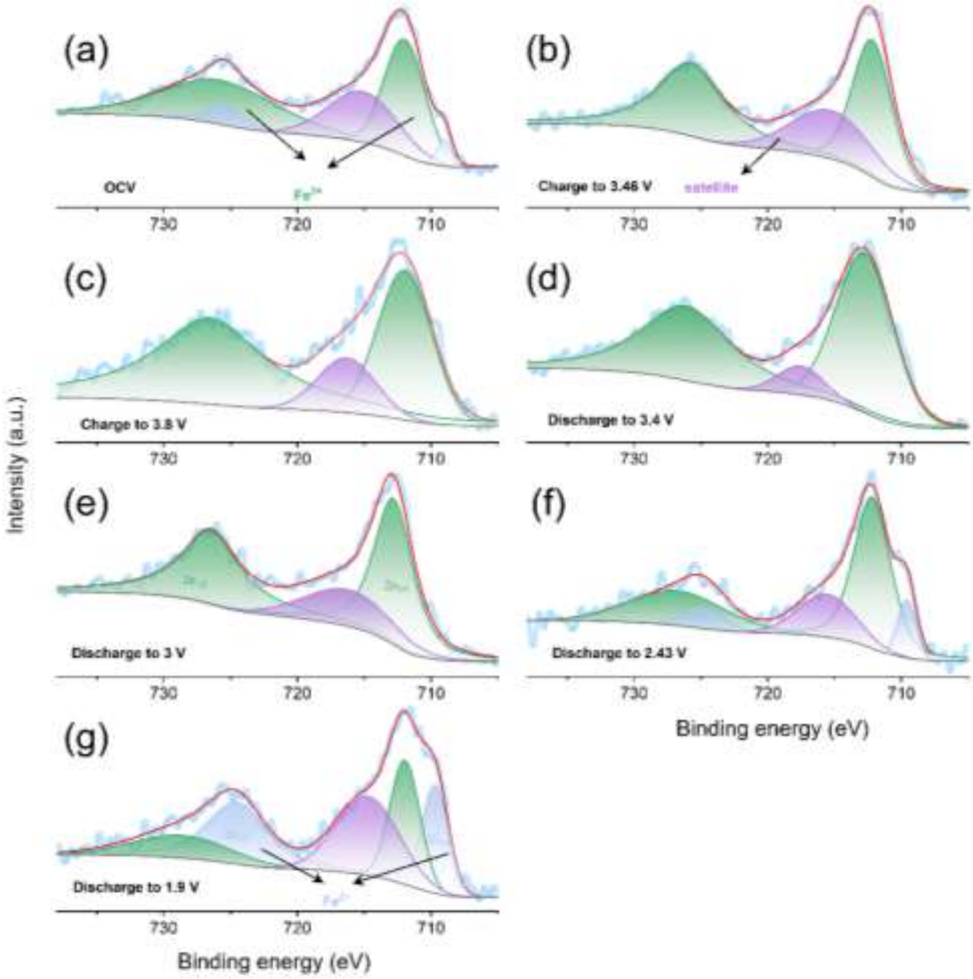

Figure S4. (a-g) The spectrum of Fe 2p XPS at different states of voltage, corresponding to the point of a-g in Figure 4e.

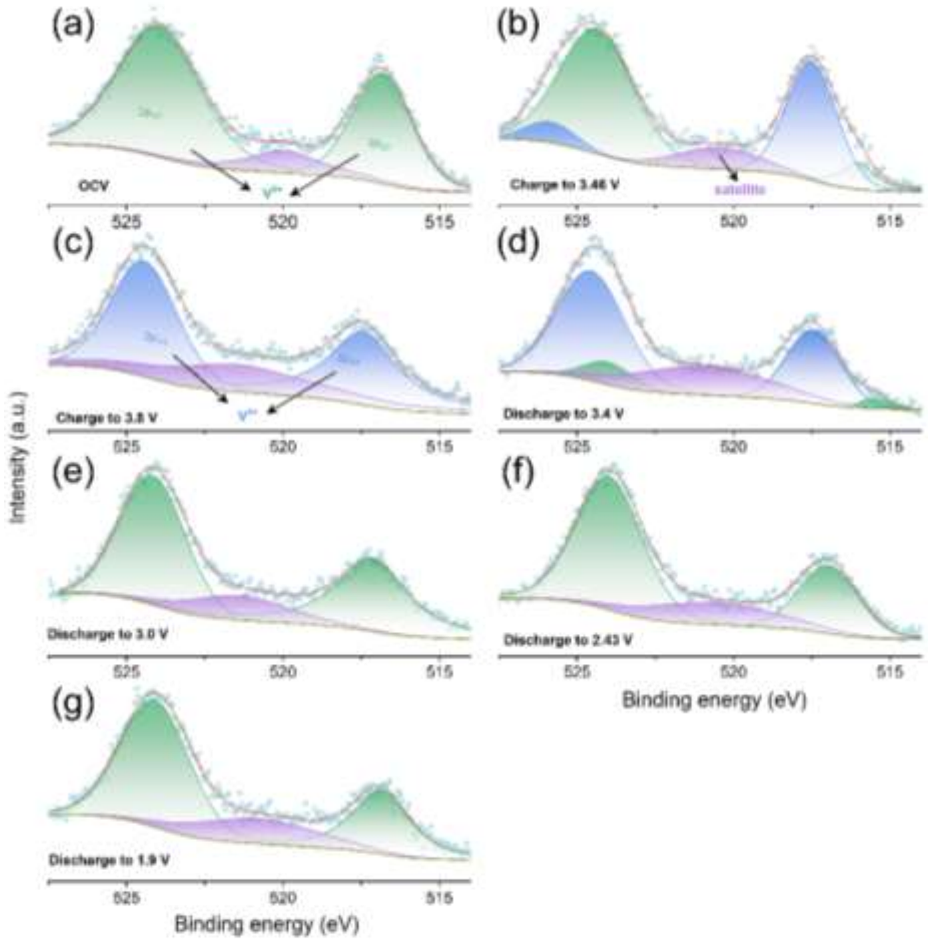

Figure S5. (a-g) The spectrum of V 2p XPS at different states of voltage, corresponding to the point of a-g in Figure 4e.

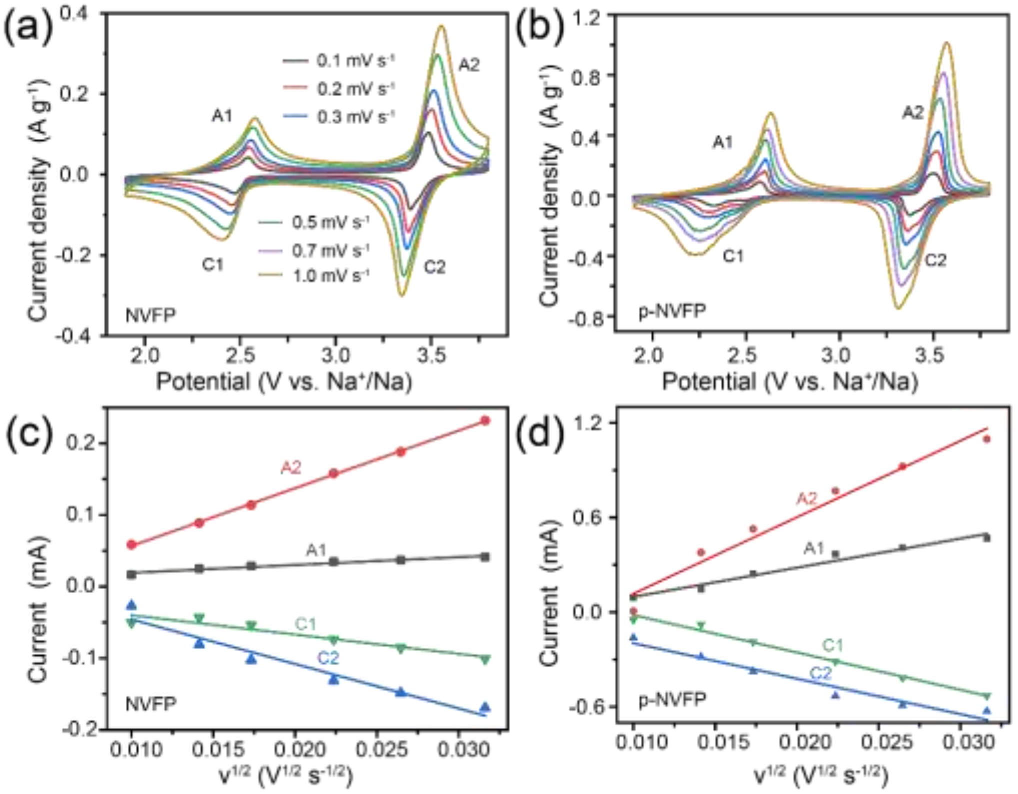

Figure S6. CV curves at various scan rates and corresponding linear fitting between the I_p and $\upsilon^{1/2}$ of (a, c) NVFP and (b, d) p-NVFP half cells.

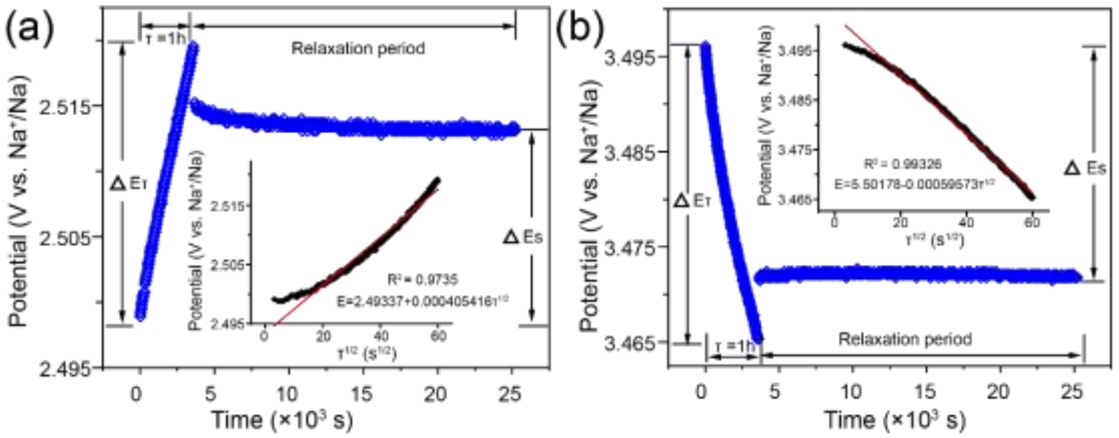

Figure S7. Single GITT titration process during galvanostatic charge and discharge of p-NVFP half cells the relationship between τ and E during (a) charging process and (b) discharge process (the inset is the linear fitting of E and $\tau^{1/2}$).

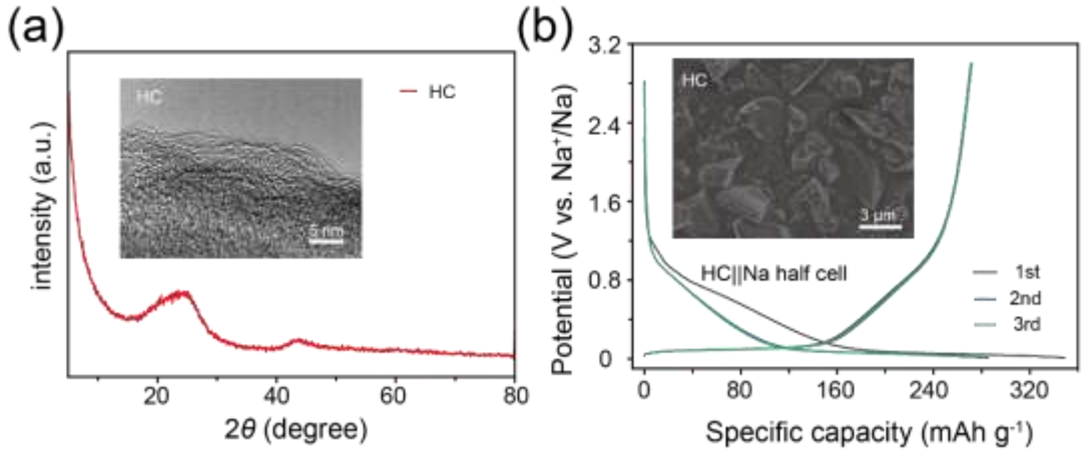

Figure S8. (a) XRD of HC sample, the inset is its TEM image; (b) Charging/discharging profile of the first three cycles for HC||Na half cells, the inset is the SEM image of HC.

Table S1. Electronic conductivity comparison of NVFP and p-NVFP.

**Pressure (MPa)**	**Electronic conductivity (mS cm^{-1})**	
	NVFP	p-NVFP
0	1.12026×10^{-11}	4.48689×10^{-11}
2	8.99908×10^{-12}	4.38298×10^{-11}
4	8.55432×10^{-12}	4.52764×10^{-11}
6	9.16955×10^{-12}	4.5972×10^{-11}
8	7.04501×10^{-12}	4.44444×10^{-11}
10	5.68894×10^{-12}	5.13403×10^{-11}
12	4.13068×10^{-12}	5.05095×10^{-11}
14	2.98273×10^{-12}	5.09075×10^{-11}
16	5.4409×10^{-12}	5.43428×10^{-11}
18	3.50825×10^{-12}	5.33808×10^{-11}
20	3.32681×10^{-12}	4.81975×10^{-11}
Average value	6.36808×10^{-12}	4.84609×10^{-11}

Table S2. The comparison of electrochemical performance for HC||p-NVFP full cells with other state-of-the-art reported NASICON materials in sodium-ion full cells.

Label	Full cell anode// cathode	Electrolyte	Voltage (V)	Cycling performance (Capacity retention, cycles, rate)	References
j	HC||p-NVFP	1M $NaClO_4$ in PC:EC (1:1) with 5 vol% FEC	1.6~3.8	92%, 500, 2 C	This work
i	PPy-coated Fe_3O_4||$Na_4Fe_3(PO_4)_2(P_2O_7)$/C	1M $NaClO_4$ in PC:EC (1:1) with 5 vol% FEC	0.1~4.0	76.9%, 500, 100 mA g^{-1}	[1]
h	hard carbon||$Na_4V_2(PO_4)_3$	1 M $NaPF_6$ dissolved in diglyme	2.0~3.8	70%, 100, 0.5 C	[2]
g	HC||$Na_3Mn_{0.95}Sc_{0.05}Ti(PO_4)_3$	1M $NaClO_4$ in PC:EC (1:1) with 5 vol% FEC	1.3~4.2	82%, 200, 1 C	[3]
f	FBO@ C||NFPP@rGO	1M $NaPF_6$ in EMC-PC (1:1 wt) with 5 vol% FEC	-	76%, 250, 1 C	[4]
e	HC||NFPP@C@rGO	1M $NaClO_4$ in DEC-EC (1:1 vol) with 5 vol% FEC	1.7~4.3	70.1%, 200, 1 C	[5]
d	HC||NMF-NVP/NC	1M $NaClO_4$ in EC:DEC (1:1) with 5 vol% FEC	1.5~3.7	60%, 200, 1 C	[6]
c	HC||NFVP/MP	1.0 M $NaClO_4$ in PC/FEC (95 : 5 volume ratio)	1.0~3.8	76%, 100, 0.5 C	[7]
b	NVP/CGO||NVP/CGO	1M $NaClO_4$ in DMC:EC (1:1 wt) with 5 wt % FEC	1~2.4	75%, 100, 1C	[8]

a	o-$Na_2C_6H_2O_6$‖NVP@ C/MWCNTs	1M $NaClO_4$ in EC:DMC (1:1) with 5 vol% FEC	-	67.8%, 100, 0.05 C	[9]

Reference

[1] M. Chen, W. Hua, J. Xiao, D. Cortie, W. Chen, E. Wang, Z. Hu, Q. Gu, X. Wang, S. Indris, S. L. Chou, S. X. Dou, *Nat Commun* **2019**, 10, 1480.

[2] Y. Liu, X. Wu, A. Moeez, Z. Peng, Y. Xia, D. Zhao, J. Liu, W. Li, *Adv. Energy Mater.* **2022**, 13, 2203283.

[3] K. Chen, Q. Shi, Y. Wang, X. Li, Y. Jiang, H. Xu, S. Guo, L. Zhao, C. Dai, *Colloids and Surfaces A: Physicochemical and Engineering Aspects* **2023**, 662, 130996.

[4] Y. Cao, X. Cao, X. Dong, X. Zhang, J. Xu, N. Wang, Y. Yang, C. Wang, Y. Liu, Y. Xia, *Adv. Funct. Mater.* **2021**, 31, 2102856.

[5] J. Gao, Y. Tian, Y. Mei, L. Ni, H. Wang, H. Liu, W. Deng, G. Zou, H. Hou, X. Ji, *Chem. Eng. J.* **2023**, 458, 141385.

[6] L. Zhao, H. Zhao, J. Wang, Y. Zhang, Z. Li, Z. Du, K. Swierczek, Y. Hou, *ACS Appl. Mater. Interfaces* **2021**, 13, 8445.

[7] L. M. Zhang, K. Cao, S. Wang, F. Chen, J. M. Dong, N. Q. Ren, Y. X. Li, Z. Y. Wen, C. H. Chen, *Nanoscale* **2022**, 14, 15640.

[8] Y. Zhang, M. Wu, R. Zhang, Y. Huang, *ACS Appl. Energy Mater.* **2020**, 3, 2867.

[9] L. Chen, Z. Zhong, S. Ren, D.-M. Han, *Energy Technol.* **2020**, 8, 1901080.
